# CXCR4: A new player in vestibular schwannoma pathogenesis

**DOI:** 10.18632/oncotarget.24119

**Published:** 2018-01-10

**Authors:** Maria Breun, Alexandra Schwerdtfeger, Donato Daniel Martellotta, Almuth F. Kessler, Jose M. Perez, Camelia M Monoranu, Ralf-Ingo Ernestus, Cordula Matthies, Mario Löhr, Carsten Hagemann

**Affiliations:** ^1^ Department of Neurosurgery, University Hospital Würzburg, 97080 Würzburg, Germany; ^2^ Department of Neuropathology, University of Würzburg, Institute of Pathology, 97080 Würzburg, Germany; ^3^ Comprehensive Cancer Center (CCC), Mainfranken, Würzburg

**Keywords:** vestibular schwannoma, CXCR4, CXCL12 chemokine, tumor microenvironment

## Abstract

**Background:**

CXCR4 is a chemokine receptor that recruits blood stem cells and increases tumor cell growth and invasiveness. We examined CXCR4 expression in vestibular schwannomas (VS) from patients with and without neurofibromatosis type 2 (NF2) and correlated the levels with the patients’ clinical characteristics. The aim was to determine whether CXCR4 can be used as a prognostic marker and as a target for systemic therapy.

**Results:**

Overall, CXCR4 mRNA levels were 4.6-fold higher in VS versus control; the levels were 4.9-fold higher in NF2 patients and 4.2-fold higher in sporadic VS patients. IHC and WB showed heterogeneous protein expression, and CXCR4 was expressed mainly in S100-positive Schwann cells. There was no correlation between the CXCR4 protein levels and tumor extension. However, there was a trend towards correlation between higher expression levels and greater hearing loss.

**Materials and Methods:**

CXCR4 mRNA and protein levels were determined in VS samples (*n* = 60); of these, 30 samples were from patients with NF2. Healthy nerves from autopsies served as controls. CXCR4 mRNA levels were measured by PCR, and protein levels were measured by immunohistochemistry (IHC) and Western blotting (WB). Tumor extension and hearing loss were categorized according to the Hannover Classification as clinical parameters.

**Conclusions:**

CXCR4 mRNA was overexpressed in VS relative to healthy vestibular nerves, and there was a trend towards higher CXCR4 expression levels being correlated with greater functional impairment. Thus, CXCR4 may be a prognostic marker of VS, and CXCR4 inhibition has potential as a systemic approach for the treatment of VS.

## INTRODUCTION

Vestibular schwannomas are benign nerve sheath tumors of the vestibulocochlear nerve that are composed entirely of neoplastic Schwann cells [1, 2]. These tumors may arise sporadically, but they are also associated with a rare (1:33,000) genetic disorder, neurofibromatosis type 2 (NF2). In NF2, tumors like schwannomas, meningiomas, and ependymomas develop due to the loss of the NF2 gene on chromosome 22. The NF2 gene encodes merlin, a tumor suppressor protein [3, 4], and vestibular schwannoma is the hallmark tumor of NF2. In NF2, tumors usually grow bilaterally, and compared to sporadic schwannomas, they grow faster, have a higher recurrence rate, and are much more adherent to the cranial nerves and the brainstem [5]. Accordingly, NF2-associated vestibular schwannomas are the more aggressive tumor entity. Sole surgery is not a long-lasting solution, as it is often associated with persistent cranial nerve deficits. Thus, efficacious systemic therapy is urgently needed.

Merlin is a FERM (4.1 protein/ezrin/radixin/moesin) protein that links the cell membrane and the cytoskeleton. Merlin is activated by intercellular adhesion and by attachment to the extracellular matrix [6]. Phosphorylation of serine 518 in response to integrin or CD44-mediated signaling switches merlin from its active form to an inactive state. Merlin’s loss of function results in the activation of two main signaling pathways, the Ras/Raf/MEK pathway and the PI3K/Akt/mTOR pathway, which inhibits apoptosis and results in a higher rate of cell survival and proliferation [3, 7, 8]. Other pathways, like the Hippo pathway and the VEGF-mediated signaling pathway, are also influenced by Merlin’s loss of function [3, 6]. Indeed, therapeutic Bevacizumab is used to target VEGF overexpression by vestibular schwannomas in NF2 vestibular schwannoma [3]. In terms of decreasing tumor size or even maintaining a stable disease state, there is currently no effective systemic treatment option available for vestibular schwannomas. There is a great need to identify suitable molecular therapeutic targets, especially for patients with NF2 [3, 9].

Chemokines are an important part of the tumor environment [9], which, in addition to NF2 loss in Schwann cells, is essential for tumor development. As a new therapeutic target, chemokines could represent a new approach to vestibular schwannoma treatment.

Chemokine receptor-4 (CXCR4), a 40-kDa G protein-coupled receptor of the chemokine receptor subfamily, consists of seven transmembrane domains. The CXCR4 gene is located on chromosome 2, and the CXCR4 protein was initially found to regulate leucocyte trafficking [10, 11]. CXCR4 plays roles in the homing and recruitment of stem cells, progenitor cells, and immune cells, and it is important for the development of the nervous, hematopoietic, and cardiovascular systems during embryogenesis [10, 11, 13]. However, it is also involved in some pathological processes, including infection, autoimmune disease, and cancer [10, 14]. CXCR4 is overexpressed in blood, breast, prostate, lung, and colon cancer, as well as in neuroblastoma and peripheral nerve sheath tumors [11, 15]. CXCL12, also called SDF1, is the only known ligand for CXCR4, and helps direct metastatic cells to CXCL12-expressing organs. CXCL12 binding to the CXCR4 receptor results in activation of the Ras/Raf/MEK and the PI3K/Akt/mTOR signaling pathways, which are also the main pathways activated by the loss of Merlin [11]. These characteristics suggest that CXCR4 could be involved in vestibular schwannoma development, since it increases tumor cell growth, invasiveness, and metastasis in many tumor types.

Few studies have examined the roles of chemokines in vestibular schwannoma [16, 17]. In those studies, CXCL12 overexpression of CXCL12 and its receptor, CXCR4, were found in *n* = 6 tumor samples. However, there are no data on CXCR4 expression in vestibular schwannomas in patients with or without NF2, nor has CXCR4 expression been correlated with other clinical data. CXCR4 is overexpressed in several tumor types, where it accelerates tumor growth and invasiveness [11], but it can be blocked by CXCR4-specific inhibitors [11, 12]. We hypothesized that CXCR4 might be an interesting therapeutic target for the treatment of vestibular schwannoma. Therefore, we examined the expression and spatial distribution of CXCR4 in sporadic and NF2-associated tumor tissue and evaluated its potential role as a prognostic marker for vestibular schwannoma.

## RESULTS

### Clinical and MRI data

Tumor extension was greater in patients with NF2-associated vestibular schwannoma than in patients with sporadic vestibular schwannoma. In NF2 patients, there were 5 samples from small tumors (T3A or smaller according to the Hannover Classification) and 25 from large tumors (T3B or larger). In patients with sporadic tumors, there were 10 samples from small tumors (T3A or smaller and 20 samples from large tumors (T3B or larger).

Hearing function was worse in patients with NF2-associated vestibular schwannoma than in patients with sporadic vestibular schwannoma. NF2 patients showed good hearing function (H1/2) in 10 cases, moderate hearing (H3/4) in 12 cases, and at least functional deafness (H5/6) in 12 cases according to the Hannover Classification. Patients with sporadic vestibular schwannoma showed good hearing (H1/2) in 8 cases, moderate hearing (H3/4) in 14 cases, and at least functional deafness (H5/6) in 8 cases (Table 1).

**Table 1:** Clinical features of patients with NF VS and sporadic VS. Tumor extension and hearing function according to the Hannover classification are shown along with the respective histological subtype

Tissue	Tumor growth dynamic	Tumor extension		Hearing function			Histological type^*^		
		≤ T3A	≥ T3B	H1/2	H3/4	H5/6	A	B	A/B
**NF VS**	s.p.	4	11	4	7	4	9	0	4
	r.p.	1	14	6	1	8	7	1	5
**Sporadic VS**	s.p.	4	12	3	8	4	9	1	5
	r.p.	6	8	5	6	4	9	2	4

Abbreviations: s.p.= slow progression, r.p.= rapid progression, NF = neurofibromatosis type 2, NF VS = NF-associated with vestibular schwannoma, sporadic VS = sporadic vestibular schwannoma (not associated with NF).

^*^The histological Antoni type was not known for all cases.

### CXCR4 mRNA expression in vestibular schwannomas

**Figure 1:**
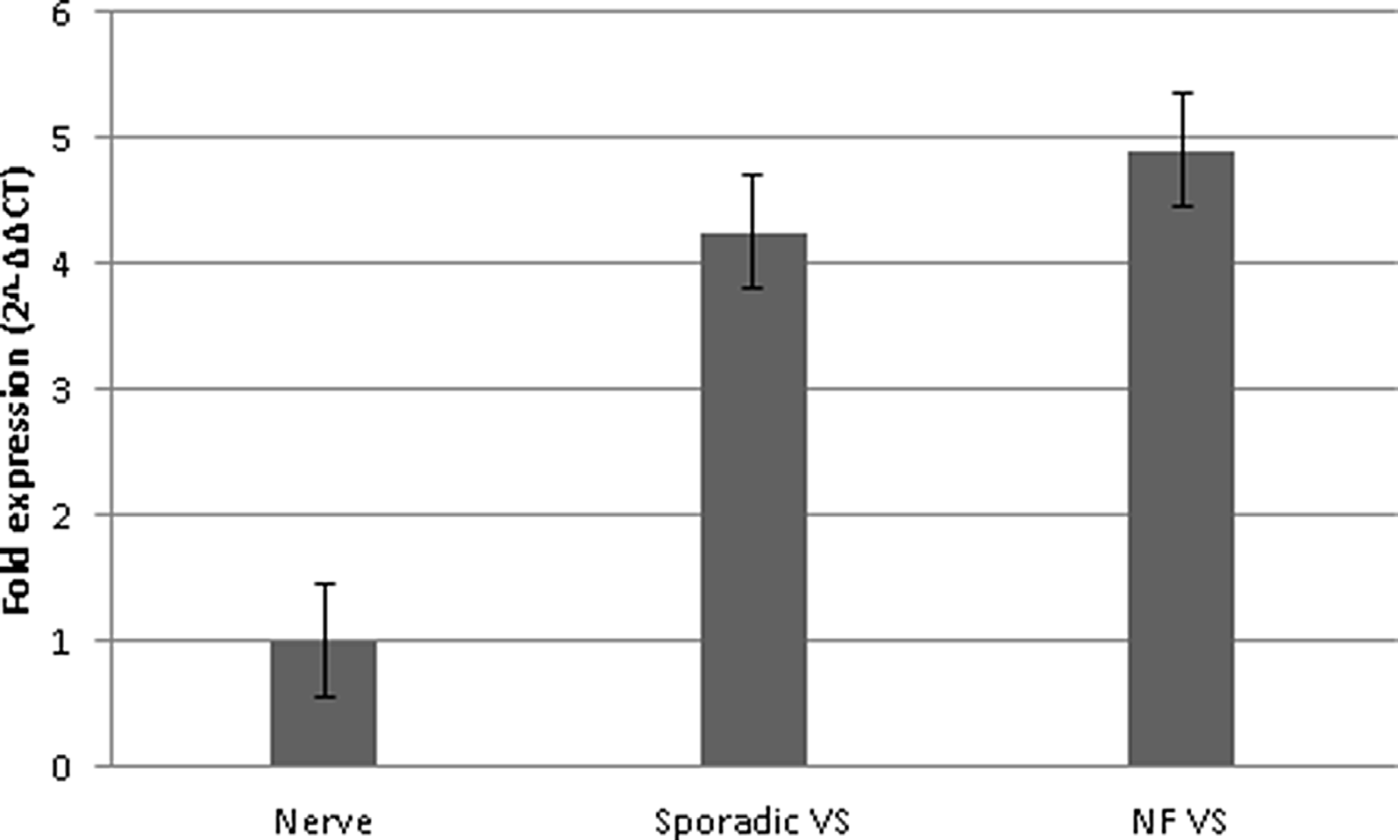
CXCR4 mRNA expression in the vestibular schwannoma subgroups CXCR4 mRNA expression was analyzed by qPCR using the 2^-∆∆CT^ method in sporadic vestibular schwannoma (sporadic VS, *n* = 30), in NF2-associated vestibular schwannoma (NF VS, *n* = 30), and in healthy sensory nerves (Nerve, *n* = 10). Error bars indicate the SEM.

Vestibular schwannomas showed 4.6-fold higher CXCR4 mRNA expression than control samples (Figure 1). In sporadic vestibular schwannomas (mean patient age, 51 years), expression was 4.25-fold higher, and in NF2-associated vestibular schwannomas (mean patient age, 33 years), expression was 4.9-times higher compared to the control group (mean patient age, 57 years). The difference in CXCR4 expression between these subgroups was not statistically significant. Tumor growth patterns prior to surgery and tumor extension at the time of surgery did not correlate with the CXCR4 mRNA expression level (data not shown and Table 1).

**Figure 2:**
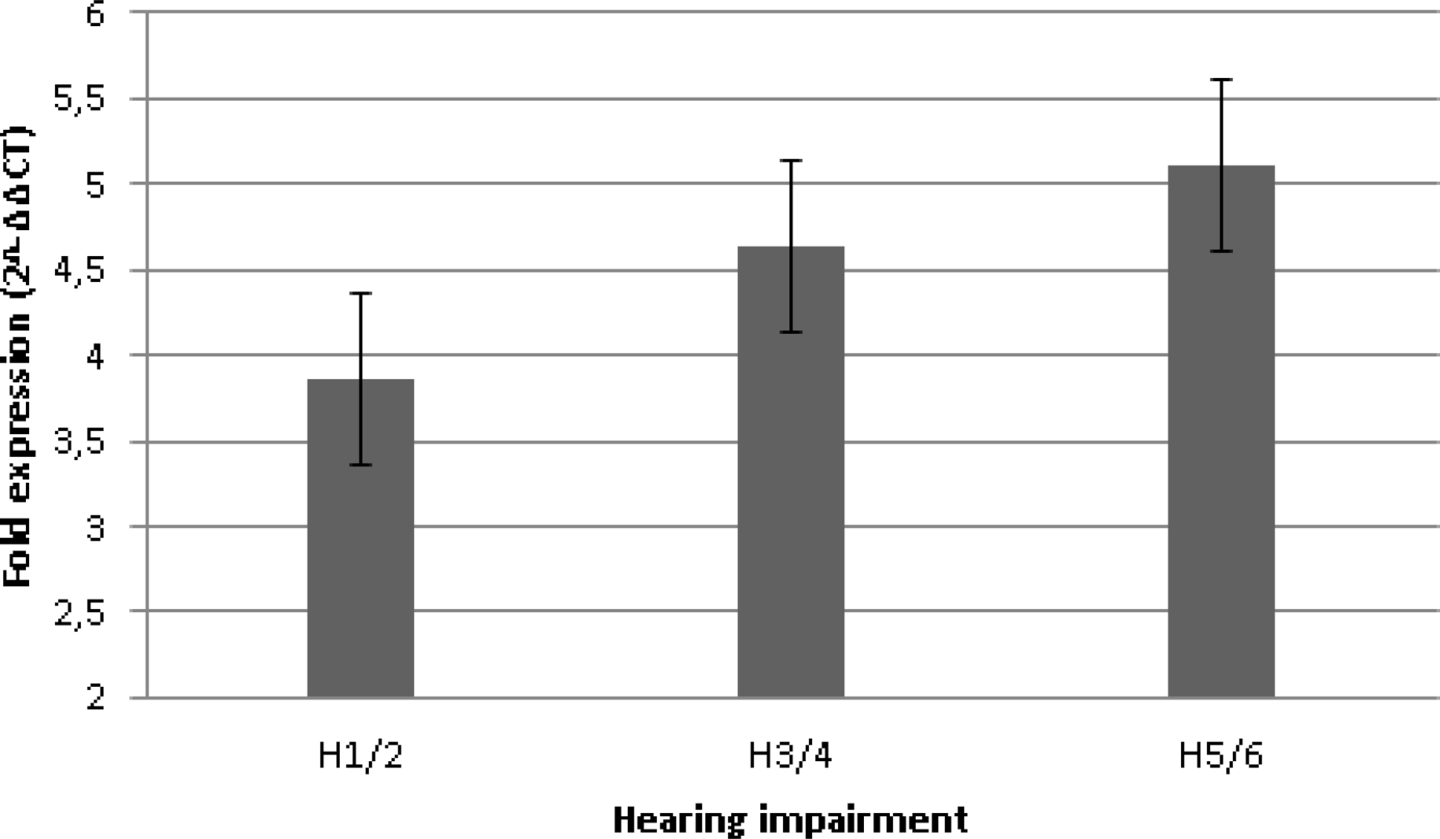
CXCR4 mRNA expression and hearing impairment Hearing impairment caused by vestibular schwannoma was categorized according to the Hannover Classification. H1 indicates a maximum hearing loss of 20 dB, H2 a loss of 40 dB, H3 a loss of 60 dB, H4 a loss of 80 dB, H5 a loss of 100 dB, and H6 a loss greater than 100 dB. The Pearson correlation coefficient was not significant.

CXCR4 mRNA expression was 3.9-fold higher in patients with slight hearing loss (H1/2), 4.6-fold higher in patients with moderate hearing impairment (H3/4), and 5-fold higher in patients with severe hearing loss or deafness (H5/6) compared to the control group. The differences were not statistically significant (Figure 2), but greater hearing impairment showed a trend towards correlation with higher CXCR4 expression levels.

### CXCR4 protein expression in vestibular schwannomas

**Figure 3:**
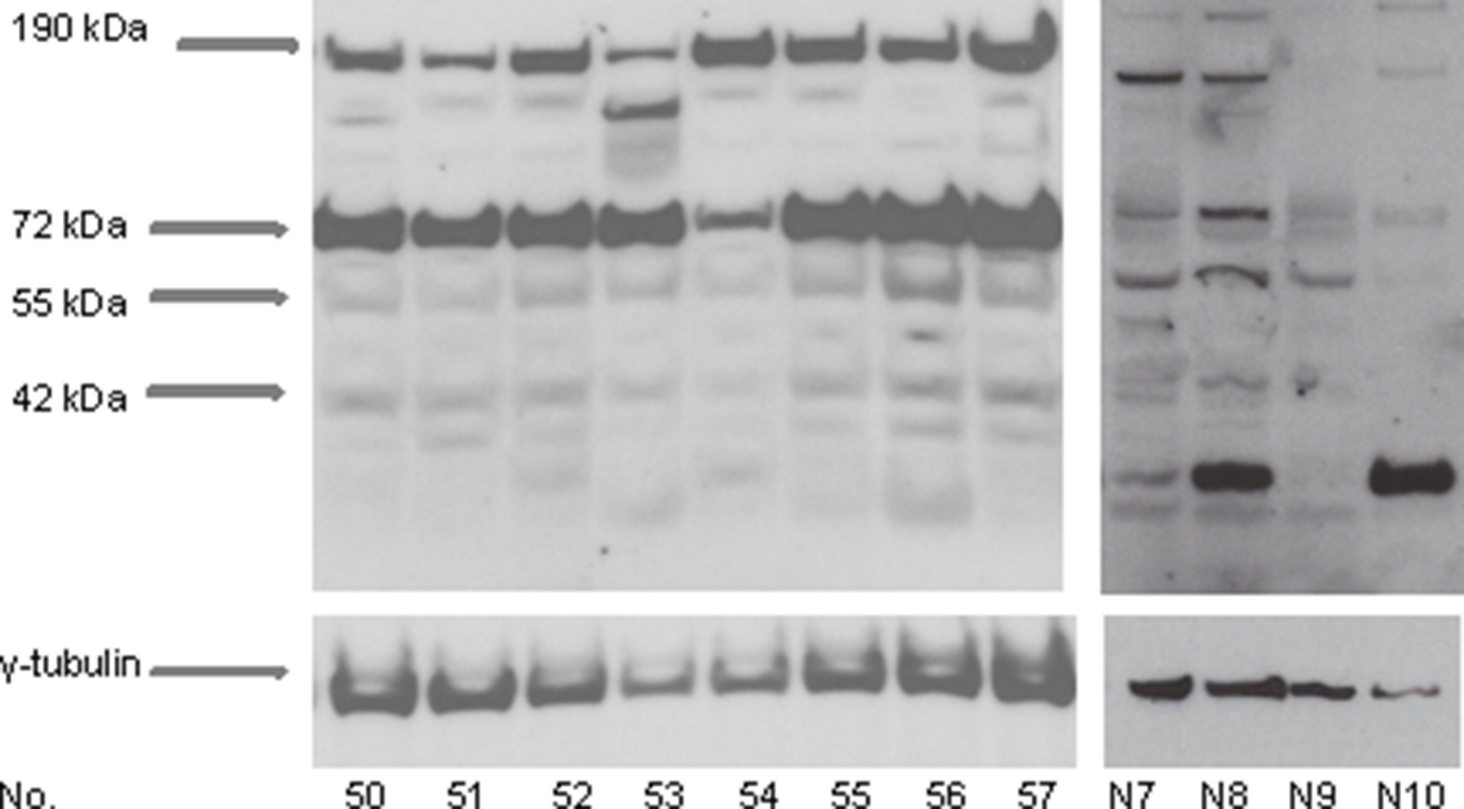
CXCR4 protein expression in vestibular schwannomas Protein lysates were isolated from vestibular schwannoma samples and subjected to Western blotting analysis. Specific antibodies detected at least four CXCR4 isoforms of approximately 190 kDa, 72 kDa, 55 kDA, and 42 kDa (arrows), reflecting the structural heterogeneity of CXCR4 in tumors. γ-tubulin served as a loading control. Results are shown for 8 representative tumors (No. 50–57) of the total sample (*n* = 60) and for 4 representative nerves (No. N7–N10) for the control group (*n* = 10).

**Figure 4:**
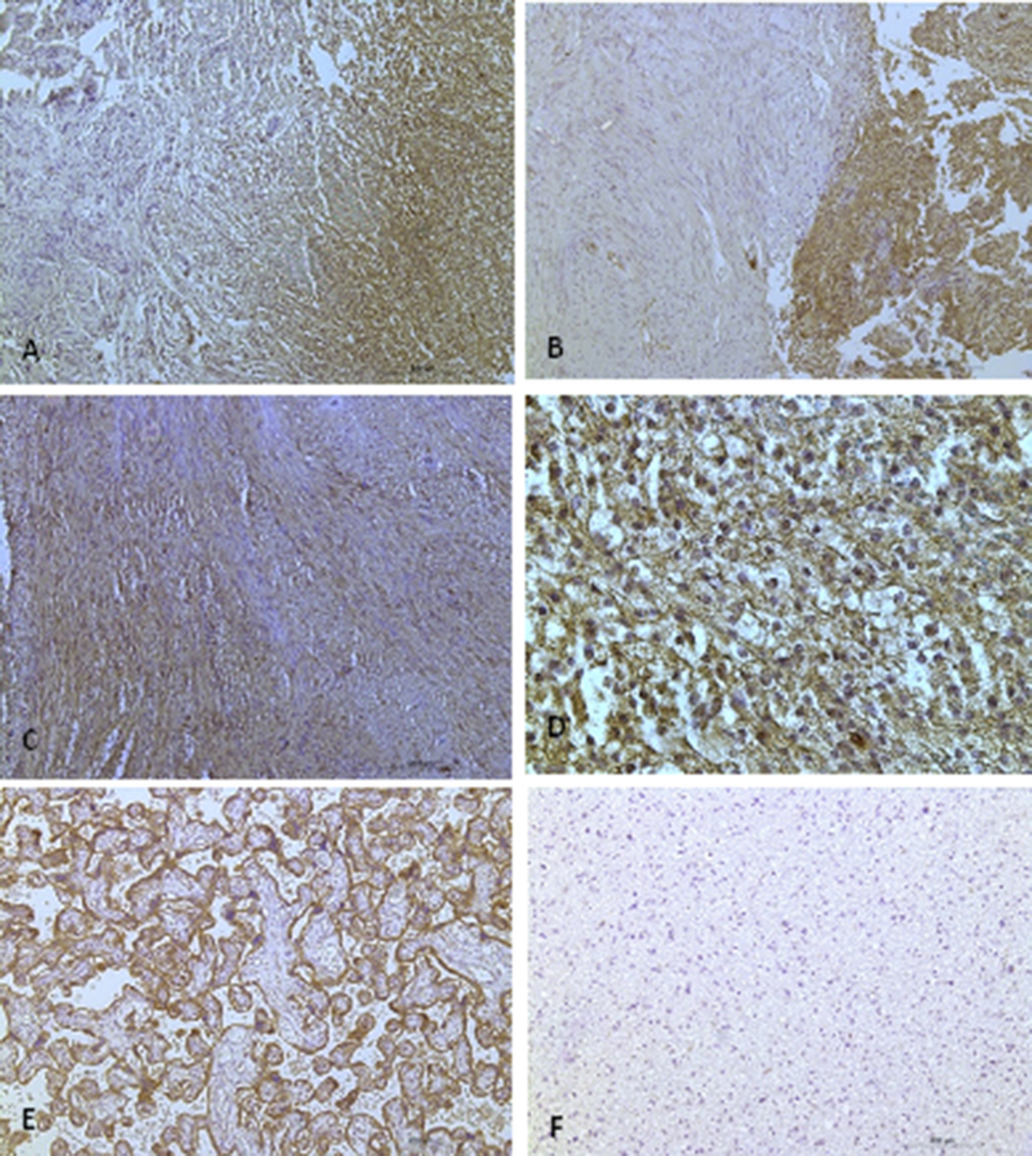
CXCR4 protein expression in vestibular schwannomas (**A** and **B**) Antoni type A regions show weak staining, while Antoni type B regions are strongly stained. (A) is a sporadic vestibular schwannoma, and (B) is an NF2-associated vestibular schwannoma. (**C**) There is strong staining in Antoni type A regions. (**D**) 40X magnification reveals CXCR4 protein expression at the cell membrane (scale bar, 50 µm). (**E**) Placental tissue served as a positive control and (**F**) astrocytoma tissue was used as a negative control. (A–C, E, F): Scale bar, 200 μm.

Western blotting analysis of proteins from vestibular schwannomas revealed four CXCR4 isoforms of approximately 190 kDa, 72 kDa, 55 kDa, and 42 kDa. The 190 kDa and 72 kDa isoforms showed the strongest expression (Figure 3). CXCR4 protein expression was also detected by immunohistochemical analysis of sections of vestibular schwannomas. Since it is a transmembrane receptor, CXCR4 was detected mainly on the membranes of Schwann cells (Figure 4).

In the mixed Antoni A/B cases (*n* = 18), regions with high cellular density in a spindle-shaped arrangement (i.e. regions with Antoni A characteristics) usually showed weak CXCR4 staining. In contrast, CXCR4 was strongly expressed in areas where there was a loose meshwork of gelatinous and microcystic tissue (regions with Antoni B characteristics). However, this association with the Antoni patterns was only observed in 11 of the 18 mixed type (Antoni A/B) cases, and thus it was not a general finding. In samples with only Antoni type A or B tissue, there was no association between the histoarchitecture and CXCR4 expression on the membranes (Figure 4). Furthermore, the strength of the immunohistochemical staining was not correlated to tumor size, hearing function, or NF2.

**Figure 5:**
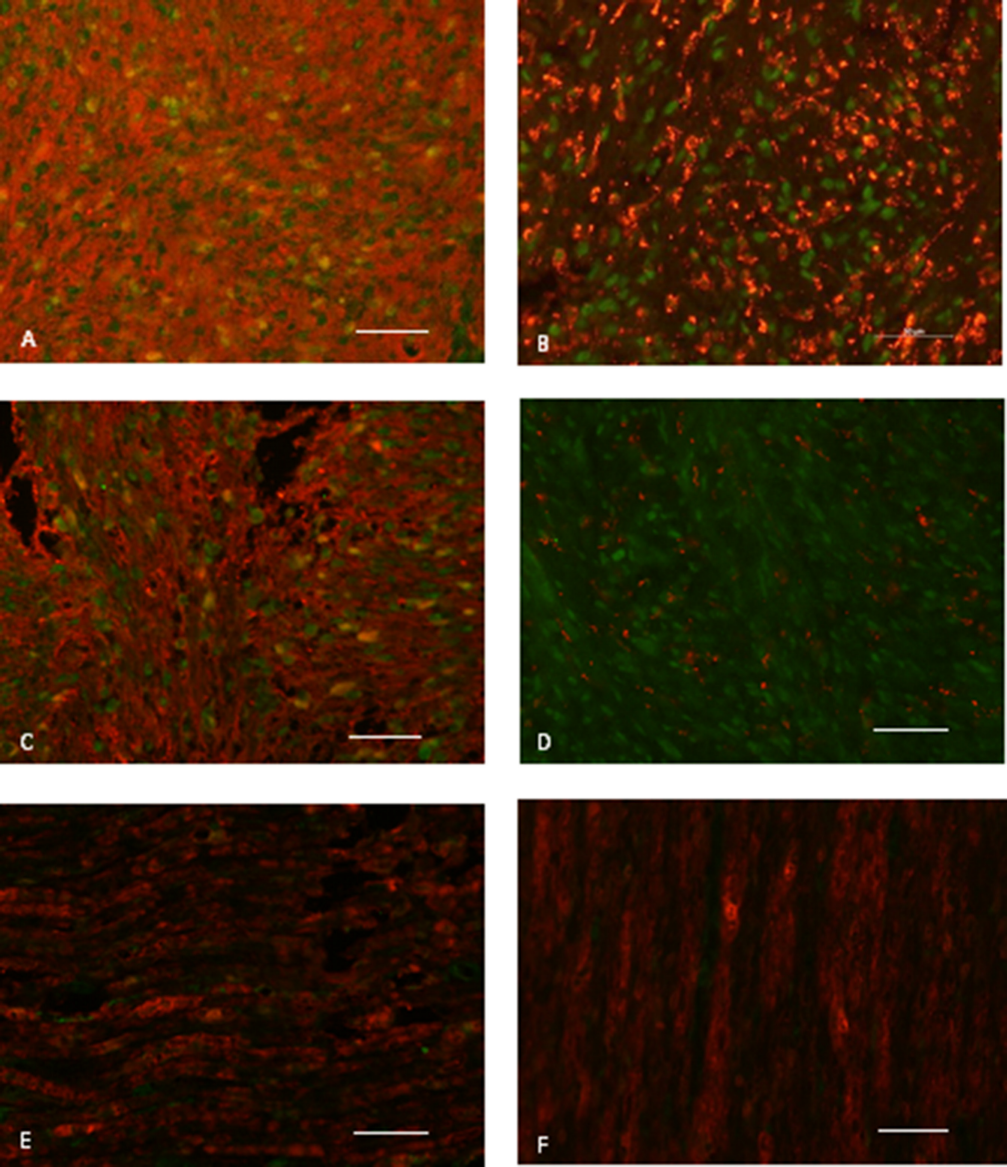
CXCR4 and CXCL12 expression on paraffin-embedded sections of a representative vestibular schwannoma sample (**A**) CXCR4 (green) co-localizes with the Schwann cell/tumor marker S100 (red). (**B**) CXCR4 (green) does not co-localize with CD68 (red), a macrophage marker, indicating that these cells do not express CXCR4. (**C**) CXCL12 (green) co-localizes with S100 (red) but (**D**) not with CD68 (red) in the main part of the tumor. E and F) Although healthy vestibular nerves obtained from autopsies show positive S100 (red) staining, they show no significant CXCR4 or CXCL12 expression (green). Scale bar, 50 µm.

Double immunofluorescence staining of vestibular schwannomas for CXCR4 and the Schwann cell protein S100 revealed co-localization of these proteins. In contrast, CXCR4 and the macrophage protein CD68 showed no co-localization. Thus, CXCR4 was expressed in the Schwann cells of vestibular schwannomas. CXCL12, the CXCR4 ligand, was also mainly expressed in S100-positive cells, whereas tissue from control samples showed very low CXCR4 and CXCL12 expression. CD68-positive cells were barely detectable in the control group (Figure 5).

## DISCUSSION

The present investigation demonstrates for the first time in a larger cohort that CXCR4 is overexpressed in pure vestibular schwannomas with and without NF2 and could therefore play a role in the pathogenesis of these tumors. Four isoforms of the protein were detected in the tumors, and immunostaining revealed heterogeneous distribution of the protein within vestibular schwannoma tissue, especially in tissue showing an Antoni A/B pattern. There was no correlation between either tumor extension or tumor growth rate and the CXCR4 expression level, but there was a trend towards a correlation between greater hearing impairment and higher CXCR4 expression. CXCR4 and CXCL12 were expressed mainly in S100-positive tumor cells and not in tumor-associated macrophages.

NF2-associated vestibular schwannomas often develop in younger patients, are more invasive, and are faster growing [5]. CXCR4 expression was not significantly different in NF2-associated vestibular schwannomas than in sporadic vestibular schwannoma. Although tumor extension is the strongest predictor of hearing impairment in vestibular schwannoma [18], there is sometimes a mismatch between tumor size and hearing level. For example, some patients with large tumor extension still have adequate hearing, while others with small tumors have distinct hearing impairment [18]. Although vestibular schwannomas are benign tumors, they can invade the surrounding neural tissues. An invasive growth pattern due to CXCR4 overexpression could explain why hearing impairment does not always correlate with tumor size. Indeed, we found no correlation between CXCR4 expression and tumor extension. We conclude that CXCR4 is not a marker for the tumor extension but may be relevant for tumor invasiveness, as reflected by hearing impairment. Known mechanisms are Calcium release, ERK1/2 phosphorylation, IP3/Akt activation, and MAP signaling activation are all downstream of CXCR4 activation and lead to greater invasiveness and to the proliferation of diverse tumor cells [19]. In glioblastoma, CXCR4 expression can be detected by non-invasive (68)Ga-Pentixafor-PET/CT [20]. In the future, routine assessment of CXCR4 expression might be an option for evaluating the risk of individual patients for hearing impairment.

As Carlisle et al. 2009 described for neuroblastoma, we detected different isoforms of CXCR4 in vestibular schwannomas by Western blot analysis in the present series. These isoforms reflected the high structural heterogeneity of CXCR4. In neuroblastoma, CXCR4 surface expression classes can be identified according to the expression levels of different isoforms [21]. Two major isoforms of approximately 38 kDa and 45 kDa, which correspond to the 42 kDa isoform detected in our study, were reported to be native and glycosylated forms of a protein. Furthermore, there were found exclusively in neuroblastoma cell lines that showed low expression of CXCR4 on the cell surface. The 55 kDa, 67 kDa, and 87 kDa isoforms correlated with high expression of CXCR4 on the cell surface in neuroblastoma cell lines but were also seen in some cell lines that showed low expression levels of CXCR4 on the cell surface. Ubiquitination of CXCR4 is partially responsible for the heterogeneity we detected by Western blotting [21, 22, 23, 24].

CXCR4 inhibition is possible and well tolerated, and it is used as a therapeutic option for other solid tumors. AMD3100 (Plerixafor; Genzyme, Cambridge, MA) is commercially available and has been used in phase I and II trials against different types of cancer [11, 12, 25, 26, 27, 28]. It is a specific receptor antagonist of CXCR4. AMD3100 is well tolerated, and apart from diarrhea, vomiting, headache, and hypoesthesia, no severe side effects have been reported [29, 11]. Its use requires caution, because it was originally developed to induce progenitor/stem cell mobilization from the bone marrow for HIV treatment [30]. Subsequently, this blood stem cell mobilizing effect was exploited in leukemia therapy, and it is now in trials for different cancer types. However, changes in the blood count are a likely side effect of this cancer therapy. Since a systemic therapeutic approach is urgently needed in vestibular schwannoma, especially in NF2-associated vestibular schwannoma, CXCR4 inhibition could be a promising new option with the use of AMD3100 or other more specific CXCR4 inhibitors with fewer side effects, like BL8040 (Bioline, Luckenwalde, Germany)

## MATERIALS AND METHODS

### Tissue samples

This study analyzed 60 vestibular schwannoma tumor samples from 58 patients (32 women, 26 men; mean age, 42 years). Informed consent was obtained from the patients for the use of their tissue, and the study was approved by the local ethics committee. Immediately after surgical excision, the tumor was cut into halves: one half was cryopreserved, and the other half was fixed in formalin. Of the 60 samples, 30 were from patients with NF2, and 30 were from patients with sporadic vestibular schwannoma. All tumor samples were assessed histologically by a neuropathologist using the criteria of the World Health Organization. Antoni type A tissue is characterized by high cell density in a spindle-shaped arrangement [31], and Antoni type B tissue is characterized by a loose meshwork of gelatinous and microcystic tissue [31]. Mixed types, called Antoni A/B, are also common. The control samples (*n* = 10) included 4 sural nerves from biopsies and 6 normal vestibular nerves that were obtained from autopsies within the first 24 h after death. Their use was approved by the local ethics committee.

### Clinical parameters

Hearing function and tumor extension were estimated using the Hannover Classification [18]. Hearing function was estimated by tone and speech audiometry several days before surgery. Hearing deterioration was categorized into 6 classes in 20-dB steps: H1 is nearly normal hearing function, with a maximum of 20-dB hearing loss in tone audiometry and a corresponding result in speech audiometry, and H6 corresponds to deafness, with at least 100-dB hearing loss and no speech discrimination. The extension of the tumor in the cerebellopontine angle was determined: T1 indicates an intrameatal tumor, T2 indicates an intra- and extrameatal tumor, a T3A tumor fills the cerebellopontine cistern, a T3B tumor reaches the brain stem, a T4A tumor compresses the brainstem, and a T4B tumor dislocates the brain stem.

Tumor growth dynamics were categorized by sequential magnetic resonance imaging (MRI) performed prior to surgery. Both groups had 15 slowly growing vestibular schwannomas (growth rate by MRI of less than 2 mm per year or <2% Ki67-positive cells) and 15 rapidly growing tumors (growth rate by MRI of more than 2 mm per year or >2% Ki67-positive cells [32, 33].

### mRNA and protein extraction

Protein and mRNA were purified from 30 mg of tissue using the NucleoSpin RNA/Protein Kit (Macherey-Nagel, Düren, Germany) according to the manufacturer’s instructions. The concentrations of the isolated mRNA and of the extracted protein were measured with the Qubit 2.0 Fluorometer (Thermo Fisher Scientific, Waltham, MA, USA). mRNA was reverse-transcribed to cDNA utilizing the High-capacity RNA-to-cDNA Kit (Applied Biosystems, Waltham, MA, USA) and the T3000 Thermocycler (Biometra, Göttingen, Germany). The cDNA samples were stored at -80°C.

### Quantitative RT-PCR (qPCR)

The StepOnePlus Real-Time PCR System (Applied Biosystems) was used to analyze CXCR4 mRNA expression in vestibular schwannomas and vestibular nerve samples. The cDNA concentration was adjusted according to the sample with the lowest concentration and then mixed with TaqMan Universal Master Mix (Applied Biosystems). GAPDH-VIC PL (HS99999905_m1) was used as an internal control, and CXCR4_FAM (HS00607978) (NM_001008540.1 and NM_003467.2, amplicon length of 158 bp) assays (Applied Biosystems) were used to evaluate the relative CXCR4 expression in a duplex setting. PCR was performed for 10 min at 95°C followed by 50 cycles of 15 s at 95°C and 60 s at 60°C. All samples were run in triplicate. The data were analyzed with the 2^-∆∆CT^ method.

### Western blotting analysis

Total protein extract was mixed with 6.25 µl of sample buffer, water (16 µl probe volume), and 2.5 µl of reducing agent, incubated at 70°C for 10 min, and centrifuged for 1 min at 11000 x g. Next, 20 µl of each sample were loaded onto a 4%–12% polyacrylamide NuPage Bis-Tris gel (Invitrogen, Waltham, MA, USA), and electrophoresed for 1 h at 200 V and 120 mA using the XCell SureLock system (Invitrogen). The separated proteins were transferred to nitrocellulose membranes (Invitrogen) using the iBlot kit and system (Invitrogen) following the manufacturer´s instructions. The membrane was blocked in TBST (0.1% Tween 20) plus 5% nonfat milk powder (Roth, Karlsruhe, Germany) at room temperature for 1 h and probed with rabbit polyclonal antibody 13854 against human CXCR4 (Abcam, Cambridge, UK) at a dilution of 1:500 in TBST. The secondary antibody, goat anti-rabbit IgG-HRP (Santa Cruz Biotechnology, Dallas, TX, USA), was diluted 1:1000 in TBST. The ECL Western Blotting Analysis System (Amersham, Freiburg, Germany) was used for protein detection. The blot was stripped and incubated with γ-tubulin antibody T6557 in a dilution of 1:5000 in TBST (Sigma, Munich Germany) overnight at 4°C. The secondary antibody that was used to detect the anti-γ-tubulin antibody was an anti-mouse IgG-HRP antibody used at a dilution of 1:1000 in TBST (GE Healthcare UK Limited, Freiburg, Germany). The ECL Western Blotting Analysis System (Amersham) was used to visualize the signal.

### Immunohistochemistry

Vestibular schwannoma sections (3 µm) were cut from formalin-fixed paraffin-embedded tissue blocks and stained with anti-CXCR4 antibody (Zytomed 503-18440, Berlin, Germany) using a 1:50 dilution in dilution buffer (DCS, Jena, Germany). CXCR4 protein expression was visualized using a poly-link secondary antibody and a peroxidase kit (Dako; DCS Innovative Diagnostic Systems, Jena, Germany). Positive signals resulted in brown staining, and counterstaining was performed with hematoxylin. Immunofluorescence analysis was performed on formalin-fixed 3-µm paraffin sections. The sections were washed in xylol (2 x 10 min) and in a decreasing ethanol series (5 min each in 100%, 96%, 70%, and 50% ethanol). Sections were blocked with 10% goat serum (Life Technologies, No. 50062Z, Waltham, MA, USA) in antibody dilution buffer (DCS) at a 1:2 dilution for 20 min. Double-staining was performed with anti-CXCR4 antibody (rabbit, Abcam, ab124824) at a 1:500 dilution in dilution buffer (DCS) and anti-S100 antibody (mouse, Abcam, ab4066) at a 1:100 dilution or with anti-CD68 antibody (mouse, Dianova, DLN-14440, Hamburg, Germany) at a 1:200 dilution at 4°C overnight. The same procedure was repeated with anti-CXCL12 antibody (rabbit, Abcam, ab9797) and anti-S100 antibody or anti-CD68 antibody. Protein expression was visualized using two secondary antibodies: Cy3-anti-mouse (red, Dianova, 115-165-146, Hamburg, Germany) at a dilution of 1:100 and Cy2-anti-rabbit (green, Dianova, 111-225-144) at a dilution of 1:50 for 1 h at room temperature. Slides were mounted using Fluoroshield mounting medium (Abcam).

All immunohistochemically stained slides were analyzed using a light microscope (Leica, Wetzler, Germany). Negative control experiments were performed by staining low-grade astrocytomas, and positive control experiments were performed by staining placenta sections with primary and secondary antibodies.

### Statistical analysis

mRNA expression was analyzed using StepOne software v2.3 and ExpressionSuite Software v1.04 (Thermo Fisher Scientific). GAPDH mRNA expression was used to normalize the data. Statistical analysis was performed with GraphPad Prism 6 software (GraphPad Software, La Jolla, CA, USA). Statistical significance was determined using unpaired *t*-tests for tumor growth and extension correlation and ANOVA for hearing correlation. *P* < 0.05 was considered to be significant. Correlation was evaluated using the Pearson correlation coefficient.

## CONCLUSIONS

This study found that CXCR4 was overexpressed in both sporadic and NF2-associated vestibular schwannomas and that there was no statistically significant difference in the expression levels in these two groups. Hearing impairment showed a trend for correlation with higher CXCR4 expression levels, which could be due to the higher invasiveness of tumors that express this protein at higher levels. We conclude that CXCR4 represents a potential target for a systemic therapeutic approach, especially in NF2-associated vestibular schwannomas.

## Abbreviations

AMD3100: Test substance from AnorMED No. 3100
Akt: Protein kinase B
bp: Base pair
CCC: Comprehensive cancer center
CD44: Cell adhesion molecule 44
CD68: Cell adhesion molecule 68
cDNA: Complementary deoxyribonucleic acid
CXCR4: Chemokine receptor 4
CXCL12: Chemokine ligand 12, also known as SDF1
Cy2/3: Cyanine 2/3 (fluorescence color)
dB: Decibel
ECL: Enhanced chemiluminescence
Erk1/2: Extracellular signal-regulated kinases 1/2
FAM: Context sequence
FERM: 4.1 ezrin/radixin/moesin protein
GAPDH-VIC PL: Glyceraldehyde 3-phosphate dehydrogenase sequence
(68)Ga-Pentixafor: 68-gallium-Pentixafor tracer
HRP: Horseradish peroxidase
Hx: Hearing class according to the Hannover Classification
IgG: Immunoglobulin
IHC: Immunohistochemistry
IP3/PI3: Inositol trisphosphate
Ki67: Kiel-67; proliferation marker
MAP/MEK: Mitogen-activated protein
MRI: Magnetic resonance imaging
mRNA: Messenger ribonucleic acid
mTOR: Mechanistic Target of Rapamycin
NF2: Neurofibromatosis
NM_x: Reference sequence of the gene
PET/CT: Positron emission tomography/computed tomography
qPCR: Quantitative polymerase chain reaction
Ras/Raf: Rat sarcoma/ rapidly-growing fibrosarcoma protein
r.p.: Rapid progressive
S100: Schwann cell marker protein
SDF1: Stromal cell-derived factor 1, also known as CXCL12
SEM: Standard error of the mean
s.p.: Slow progressive
Tx: Tumor extension according to the Hannover Classification
VEGF: Vascular endothelial growth factor
VS: Vestibular schwannoma
WB: Western blot
